# Complete genome sequence of polylactic acid degrading *Rhodopseudomonas palustris* strain R1 isolated from rice field soil

**DOI:** 10.1128/mra.00814-24

**Published:** 2024-10-14

**Authors:** Lala Ito, Mana Masui, Josephine Galipon, Kazuharu Arakawa

**Affiliations:** 1 Institute for Advanced Biosciences, Keio University, Tsuruoka, Yamagata, Japan; 2 Systems Biology Program, Graduate School of Media and Governance, Keio University, Fujisawa, Kanagawa, Japan; 3 Faculty of Environment and Information Studies, Keio University, Fujisawa, Kanagawa, Japan; 4 Graduate School of Science and Engineering, Yamagata University, Yonezawa, Yamagata, Japan; University of Strathclyde, Glasgow, United Kingdom

**Keywords:** *Rhodopseudomonas palustris*, complete genome, nanopore sequencing, PLA degradation

## Abstract

*Rhodopseudomonas palustris* R1 is a phototrophic, purple, non-sulfur Gram-negative bacterium first isolated from the soils of rice fields, known for its polylactic acid degradability. Here, we present the complete circular genome sequence of this bacterium, spanning 5.32 Mbp and 4,949 protein-coding sequences.

## ANNOUNCEMENT

*Rhodopseudomonas palustris* R1 is a Gram-negative, photosynthetic, purple non-sulfur bacterium first isolated from the soils of rice fields in Japan (1). In a recent study, *R. palustris* R1 was shown to possess a polylactic acid (PLA)-degrading enzyme RPA1511_PLB, which could potentially be applied to recycling technologies (2). The draft genome sequence of this strain, consisting of 123 contigs, was published previously (3).

*R. palustris* R1 strain (JCM 2524) was purchased from the Microbe Division in Riken BioResource Research Center, received as a dry pellet. A single colony was extracted and grown over 72 hours at 30°C in an aerobic environment (AnaeroPack · MicroAero; Mitsubishi Gas Chemical Co., Inc.) with light on a M95 medium (1), shaking at 160 rpm, and harvested at an OD_600_ (Optical Density at 600nm) of around 0.3. The genomic DNA was extracted using the NucleoBond HMW DNA for high-molecular-weight DNA (Macherey-Nagel, Düren, Germany) according to the manufacturer’s instructions. A long-read sequencing library was prepared using the Rapid Barcoding Kit 24 V14 (product no. SQK-RBK114.24, Oxford Nanopore Technologies) and was sequenced with a GridION device (Oxford Nanopore Technologies) in a FLO-MIN114 flow cell. Subsequently, the reads were base-called and demultiplexed by Guppy version 6.5.7 (super-accurate mode). The quality of the long-read sequence was accessed using NanoPlot version 1.32.1 (4), resulting in a total of 790,653,524 bp consisting of 34,381 reads of N50 length of 41,666 bp. Adapters were removed with Porechop v0.2.3 (5), and reads longer than 50,000 bp (328 Mbp, about 62× coverage) were used for assembly using Canu version 2.2 (6). The resulting single contig was circularized by manually deleting the overlapping ends, following the overlapped coordinates specified by Canu. Error correction was performed using medaka v.1.11.3. Assembly quality was assessed using CheckM version 1.2.2 (7) with automatic lineage identification that resulted in f__Bradyrhizobiaceae (UID3695) with 99.44% completeness. The genome was annotated using the DDBJ Fast Annotation and Submission Tool (DFAST) version 1.2.18 (8). All software was used with default settings unless otherwise specified.

The assembled genome had a total length of 5,322,233 bp with G + C content of 65.0%, harboring 4,949 coding sequences, 6 rRNAs, and 57 tRNAs. According to the result of DFAST annotation, *phaZ* polyhydroxyalkanoate depolymerase was conserved in this species, and a BLASTP search also identified the conservation of PLA-degrading enzyme RPA1511_PLB (*e*-value = 0.0), consistent with prior evidence of the PLA degradation ability of this species. The availability of the complete genome of this species would facilitate the biotechnological applications of this species and its genetic repertoire.

## Data Availability

The genome sequences reported here were deposited in DDBJ under accession number AP035791, and the raw reads were deposited in the Sequence Read Archive (SRA) under BioProject accession number PRJNA1133237 as SRR29747273 run (http://getentry.ddbj.nig.ac.jp/getentry/na/AP035791/ https://www.ncbi.nlm.nih.gov/bioproject/PRJNA1133237 https://trace.ncbi.nlm.nih.gov/Traces/?view=run_browser&acc=SRR29747273&display=metadata).
